# Targeting of *Slc25a21* Is Associated with Orofacial Defects and Otitis Media Due to Disrupted Expression of a Neighbouring Gene

**DOI:** 10.1371/journal.pone.0091807

**Published:** 2014-03-18

**Authors:** Simon Maguire, Jeanne Estabel, Neil Ingham, Selina Pearson, Edward Ryder, Damian M. Carragher, Nicolas Walker, Sanger MGP Slc25a21 Project Team, James Bussell, Wai-In Chan, Thomas M. Keane, David J. Adams, Cheryl L. Scudamore, Christopher J. Lelliott, Ramiro Ramírez-Solis, Natasha A. Karp, Karen P. Steel, Jacqueline K. White, Anna-Karin Gerdin

**Affiliations:** 1 Wellcome Trust Sanger Institute, Wellcome Trust Genome Campus, Hinxton, Cambridgeshire, United Kingdom; 2 Department of Physiology, Development and Neuroscience, University of Cambridge, Cambridge, Cambridgeshire, United Kingdom; 3 Department of Haematology, Cambridge Institute for Medical Research, Cambridge, Cambridgeshire, United Kingdom; 4 Department of Pathology and Infectious Diseases, Royal Veterinary College, Hatfield, Hertfordshire, United Kingdom; University of South Florida College of Medicine, United States of America

## Abstract

Homozygosity for *Slc25a21^tm1a(KOMP)Wtsi^* results in mice exhibiting orofacial abnormalities, alterations in carpal and rugae structures, hearing impairment and inflammation in the middle ear. In humans it has been hypothesised that the 2-oxoadipate mitochondrial carrier coded by *SLC25A21* may be involved in the disease 2-oxoadipate acidaemia. Unexpectedly, no 2-oxoadipate acidaemia-like symptoms were observed in animals homozygous for *Slc25a21^tm1a(KOMP)Wtsi^* despite confirmation that this allele reduces *Slc25a21* expression by 71.3%. To study the complete knockout, an allelic series was generated using the *loxP* and *FRT* sites typical of a Knockout Mouse Project allele. After removal of the critical exon and neomycin selection cassette, *Slc25a21* knockout mice homozygous for the *Slc25a21^tm1b(KOMP)Wtsi^* and *Slc25a21^tm1d(KOMP)Wtsi^* alleles were phenotypically indistinguishable from wild-type. This led us to explore the genomic environment of *Slc25a21* and to discover that expression of *Pax9*, located 3′ of the target gene, was reduced in homozygous *Slc25a21^tm1a(KOMP)Wtsi^* mice. We hypothesize that the presence of the selection cassette is the cause of the down regulation of *Pax9* observed. The phenotypes we observed in homozygous *Slc25a21^tm1a(KOMP)Wtsi^* mice were broadly consistent with a hypomorphic *Pax9* allele with the exception of otitis media and hearing impairment which may be a novel consequence of *Pax9* down regulation. We explore the ramifications associated with this particular targeted mutation and emphasise the need to interpret phenotypes taking into consideration all potential underlying genetic mechanisms.

## Introduction

Knockout mice are invaluable tools for studying the function of genes both during embryonic development and in the adult. Classically, gene disruption in mice is achieved by replacing a part of the target gene with a selectable marker, e.g. a *neo* cassette, leading to constitutive ablation of the targeted gene. More contemporary approaches include the creation of conditional alleles and targeted gene traps, as well as small hairpin RNA (shRNA) and the use of lentiviral transgenesis, reviewed in [1]. The Sanger Institute Mouse Genetics Project [2] is contributing to an international effort to conduct systematic, large-scale gene function analysis in a mammalian system, through the generation of mice using targeted ES cells available from the EUCOMM and KOMP resources [3]. The typical EUCOMM and KOMP knockout first conditional ready targeted trap [tm1a(EUCOMM) and tm1a(KOMP)] is a powerful allele configured with the potential to convert to a conditional allele or a *lacZ* tagged null allele [3]. This tm1a allele was selected for pragmatic reasons, including there being no requirement for additional breeding to modify the allele, as targeted traps typically disrupt expression of the targeted gene [3], [4], and the major advantage that the allele, with full potential for conversion, would be cryopreserved for subsequent community use. However, it has previously been reported that the presence of selectable markers used for gene targeting, such as the *neo* cassette in the EUCOMM and KOMP tm1a allele, can interfere with the expression of neighbouring genes [5], [6], [7], [8], [9]. It is, therefore, important that phenotypes discovered in mice carrying the knockout first conditional ready allele, are verified using the derived deletion allele.

*SLC25A21* has previously been reported to be ubiquitously expressed in all tissues tested [10], and codes for the 2-oxoadipate mitochondrial carrier (ODC) that catalyses the movement of several substrates, primarily 2-oxoadipate and 2-oxoglutarate, across inner mitochondrial membranes [10], [11], [12]. It is suspected that ODC functions by a counter exchange mechanism, catalysing the uptake of 2-oxoadipate into the mitochondrial matrix, in exchange for internal 2-oxoglutarate [10]. The physiological role of the ODC suggests that it may be involved in the human disease 2-oxoadipate acidaemia (OMIM 204750), characterised by an accumulation of 2-oxoadipate and associated metabolites in urine [11]. This inborn error of metabolism shows clinical symptoms of intellectual disability varying from mild to severe, hypotonia, seizures, motor and developmental delay, and cerebellar ataxia [13], [14], [15], [16].

Here we report characterization of the allelic series derived from the KOMP targeted allele of *Slc25a21*. From this, three distinct observations were made. Firstly, ablation of *Slc25a21* was not found to cause symptoms of 2-oxoadipate acidaemia in mice; animals homozygous for *Slc25a21^tm1b(KOMP)Wtsi^* and *Slc25a21^tm1d(KOMP)Wtsi^* null alleles were normal for all parameters tested. Secondly, we report that *Slc25a21^tm1a(KOMP)Wtsi^* homozygous mice present with profound dental, orofacial and hearing/middle ear phenotypes caused by off-target effects on the expression of the neighbouring gene *Pax9*. Finally, the resulting novel, hypomorphic allele of *Pax9* opens the door to further studies into how *Pax9* is involved in the patterning of the palatal rugae and may represent a novel model of otitis media in the mouse. With the growing resource of knockout first conditional ready targeted ES cells created by the EUCOMM and KOMP initiatives, these results represent a timely reminder that the phenotype observed in the knockout first conditional ready mice should be verified by creating and analysing the deletion allele.

## Materials and Methods

### Ethics Statement

The care and use of all mice in this study were in accordance with UK Home Office regulations, UK Animals (Scientific Procedures) Act of 1986, and were approved by the Wellcome Trust Sanger Institute Ethical Review Committee. All efforts were made to minimize suffering including housing mice in a specific pathogen-free unit at a density of 3–5 animals per cage in polysulfone individually ventilated cages [Tecniplast Seal Safe 1284L (overall dimensions of caging: (L×W×H): 365×207×140 mm, floor area = 530 cm^2^)]. Sterilised Aspen bedding substrate and standard environmental enrichment of nestlet, cardboard tunnel, and three wooden chew blocks were provided. The light cycle was maintained at 12 h light/12 h dark with lights off at 19:30 hours and no twilight period. Room temperature was 21±2°C and humidity was regulated at 55±10%. Mice were given food and water *ad libitum* unless otherwise stated [2]. Animal welfare is our primary consideration and was assessed routinely for all mice involved in this study. Adult mice were killed by terminal anaesthesia followed by exsanguination and either cervical dislocation or removal of the heart. Embryos were killed by decapitation or cooling followed by immersion in cold tissue fixative. Experimental work was performed in accordance with the Arrive guidelines [17].

### Animals

The *Slc25a21^tm1a(KOMP)Wtsi^* mice were created by blastocyst injection of targeted ES-cell clone EPD0085_1_D04 from the NIH-funded Knockout Mouse Project (KOMP) [3], [18], [19]. Generation of mice carrying the *Slc25a21^tm1b(KOMP)Wtsi^*, *Slc25a21^tm1c(KOMP)Wtsi^* and *Slc25a21^tm1d(KOMP)Wtsi^* alleles was achieved by breeding to mice expressing *Cre* (*Hprt1^Tg(CMV-Cre)Brd^*) and/or *Flp* (*ROSA26^Fki^*) recombinases. For brevity, the allelic series will be referred to hereafter in figures as tm1a, tm1b, tm1c or tm1d. The *Slc25a21^tm1a(KOMP)Wtsi^*, *Slc25a21^tm1b(KOMP)Wtsi^*, *Slc25a21^tm1c(KOMP)Wtsi^* and *Slc25a21^tm1d(KOMP)Wtsi^* colonies were maintained on a C57BL/6N;C57BL/6-*Tyr^c-Brd^* genetic background.

Mice were fed Mouse Breeders Diet (Lab Diets 5021-3, Richmond, Indiana, USA) unless otherwise specified, including *Slc25a21^tm1d(KOMP)Wtsi^* mice processed through the Mouse Genetics Project screening protocol [2]. *Slc25a21^tm1a(KOMP)Wtsi^*, *Slc25a21^tm1b(KOMP)Wtsi^* and *Slc25a21^tm1c(KOMP)Wtsi^* mice which went through the Mouse Genetics Project screening protocol were transferred to a high-fat (21.4% fat by crude content) dietary challenge (Special Diet Services Western RD-829100, Witham, UK) at 4 weeks of age. Cages were processed randomly and different genotypes could be housed together, hence there was no pattern to the order in which animals were processed. The experimental unit throughout the study was a single mouse.

Viability at postnatal day 14 was assessed from heterozygous (het) intercross matings. Furthermore, viability and embryo morphology for *Slc25a21^tm1a(KOMP)Wtsi^* mutant mice was assessed at E14.5 and E18.5.

### Validation of targeting event

To confirm the integrity and validity of each allele studied, detailed molecular characterisation was performed using short-range, long-range and quantitative PCR strategies, as described previously [20] and detailed in File S1 and Table S1.

Genotyping for *Slc25a21^tm1a(KOMP)Wtsi^* animals was performed using a combination of three short range assays (Table S1) and the *neo* count qPCR assay (0 = wt, 1 = het, 2 = hom). Conversion to *Slc25a21^tm1b(KOMP)Wtsi^*, *Slc25a21^tm1c(KOMP)Wtsi^* and *Slc25a21^tm1d(KOMP)Wtsi^* was detected using short range PCR assays (Table S1).

Work by Santagati [21] indicated the presence of a regulatory element, named conserved non-coding sequence +6 (CNS+6), for *Pax9* within the allele design for *Slc25a21^tm1a(KOMP)Wtsi^*. A PCR assay was designed (Table S1) to amplify *Slc25a21^tm1a(KOMP)Wtsi^* samples and the resulting products sequenced to determine if the CNS+6 element was disrupted.

### Expression analysis by quantitative PCR and RNA sequencing

E13.5 embryo heads [*Slc25a21^tm1a(KOMP)Wtsi^* (wt, n = 4; hom, n = 4), *Slc25a21^tm1b(KOMP)Wtsi^* (wt, n = 4; hom, n = 3), *Slc25a21^tm1c(KOMP)Wtsi^* (wt, n = 4; hom, n = 3), and *Slc25a21^tm1d(KOMP)Wtsi^* (wt, n = 4; hom, n = 3)] were processed for expression analysis by qPCR. For *Slc25a21* a custom FAM-labelled TaqMan assay (Applied Biosystems) spanning the junction of exons 8–9, 3′ to the floxed exon (Slc25a21_E8-9_F: CTGCTTCAAAACAATGGAGATGAT, Slc25a21_E8-9_R: GGGACCAGGCCTTTGTATAAGG, Slc25a21_E8-9_M: CGGGAAGAAGGGATTT) was used. For *Pax9* a pre-designed TaqMan assay was used (Mm00440629_m1, Applied Biosystems). Details of the assays and methods used are described in File S1.

RNA sequencing was performed using 5 µg of total RNA from a subset of the above E13.5 embryo head samples [3 homozygotes and 2 wild-types for each allele (12 mutant and 8 control samples in total)] as detailed in File S1.

### Physical assessment, body composition, radiography and clinical chemistry

Homozygous mice (7M and 7F) from each of the alleles (*Slc25a21^tm1a(KOMP)Wtsi^*, *Slc25a21^tm1b(KOMP)Wtsi^*, *Slc25a21^tm1c(KOMP)Wtsi^* and *Slc25a21^tm1d(KOMP)Wtsi^*), as well as age, sex and genetic background matched controls, were analysed using the standard Sanger Institute Mouse Genetics Project phenotyping screen [2]. Assays of particular relevance are described in File S1, whilst those not part of the standard phenotyping screen are described below.

At necropsy, skulls were collected and the teeth (both upper and lower jaw) and palate were reviewed and imaged (MZ16A dissecting microscope, Leica, Wetzlar, Germany; DFC490 digital camera, Canon Powershot G5, Japan). A range of 42 additional tissues and organs from homozygous *Slc25a21^tm1a(KOMP)Wtsi^* mice (2M and 2F), and age, sex and genetic background matched controls, were fixed in 10% neutral buffered formalin (Leica Biosystems, Peterborough, UK) for 15–20 hrs and processed to wax. 5 µm sections were stained with haematoxylin and eosin then reviewed by an experienced pathologist.

Micro-CT (computed tomography) images of skulls from *Slc25a21^tm1a(KOMP)Wtsi^* mice (wt, n = 2; hom, n = 2) were collected at 18 µm resolution (SkyScan 1176, SkyScan, Kontich, Belgium).

### Auditory Brainstem Response (ABR) recordings

ABRs were recorded in *Slc25a21^tm1a(KOMP)Wtsi^* mice aged 4 weeks (wt, n = 17; het, n = 27; hom, n = 7), 8 weeks (wt, n = 20; het, n = 33; hom, n = 8), 14 weeks (wt, n = 11; hom, n = 14), and 26 weeks (wt, n = 17; hom, n = 10), using the methods described in detail in Ingham et al. [22]. In addition, mice from the *Slc25a21^tm1b(KOMP)Wtsi^* colony (hom, n = 8), *Slc25a21^tm1c(KOMP)Wtsi^* colony (hom, n = 5) and *Slc25a21^tm1d (KOMP)Wtsi^* colony (hom, n = 8) were tested at 14 weeks of age.

Anaesthetised mice were placed on a heating blanket inside a sound attenuating booth and recording electrodes inserted in the skin to record responses of the left ear. For ABR threshold determination, click (0.01 ms duration) and tone pip (6, 12, 18, 24, and 30 kHz of 5 ms duration, 1 ms rise/fall time) stimuli over a range of intensity levels from 10–95 dB sound pressure level (SPL) in 5 dB steps were presented in free-field. Averaged responses to 256 stimuli, presented at 42.2/s, were analysed and thresholds established as the lowest sound intensity giving a visually-detectable ABR response.

### Middle ear analysis

Following ABR testing, *Slc25a21^tm1a(KOMP)Wtsi^* mice aged 9 weeks (wt, n = 8; het, n = 11; hom, n = 8) were killed by cervical dislocation and the right external and middle ears were examined in detail using a dissecting microscope for any signs of malformation or inflammation, including: appearance of excess cerumen in the external ear canal; thickening, whitening, sponginess or excess vascularisation of the bulla wall; clarity and vascularisation of the tympanic membrane; presence of fluid or white inflammatory material in the middle ear cavity; and solid masses or bony outgrowths of the middle ear wall or ossicles. In addition, the left side of the head of these mice was fixed in 10% formalin for 48 hours, and decalcified in 10% EDTA diluted in phosphate buffered saline (PBS) for 10 days, dehydrated, embedded in paraffin wax, sectioned at 8 µm and stained with haematoxylin and eosin.

### Reporter gene analysis

*LacZ* reporter gene wholemount expression analysis was performed on *Slc25a21^tm1a(KOMP)Wtsi^* adults [aged 15–18 weeks (wt, n = 2; het, n = 3; hom, n = 9)] as described previously [23] and detailed in File S1.

### Bone and cartilage staining

Bone and cartilage staining of *Slc25a21^tm1a(KOMP)Wtsi^* E18.5 embryos (wt, n = 10; het, n = 27; hom, n = 12) was performed using a protocol based on the Cold Spring Harbour method for Alcian blue/Alizarin Red staining [24], and detailed in File S1.

### Data analysis and statistics

A reference range approach was used to assess continuous data, including time course. For categorical data, a Fisher's exact test was used to identify phenotypic variants. More details of both approaches are presented in File S1. Since both of the above approaches to automatically identify significant calls are known to be conservative and assess each parameter in isolation, they were complemented by a manual assessment made by a biological expert who used knowledge of events on the day, or across sexes, or between related variables, to highlight additional potentially abnormal phenotypes (Table S2).

To assess the ABR data, a one-way Kruskall-Wallis ANOVA on Ranks was used where three genotypes were assessed and Mann-Whitney rank sum test where only homozygote and wild-type animals were assessed. The qPCR gene expression results for mutant mice were compared to littermate wild-type mice using a Student's t-Test assuming two-sample unequal variance. P-values for the RNA sequencing were adjusted for multiple testing with the Benjamini-Hochberg procedure. Pre-weaning lethality was assessed using the Test for One Proportion.

## Results

### Characterisation of *Slc25a21^tm1a(KOMP)Wtsi^* homozygous mice

*Slc25a21^tm1a(KOMP)Wtsi^* mice were generated and characterised on a C57BL/6N;C57BL/6-*Tyr^c-Brd^* background by the Sanger Mouse Genetics Project. Homozygous *Slc25a21^tm1a(KOMP)Wtsi^* mice were under-represented at post natal day 14 (P14), as a result of pre-weaning lethality, with 25 homozygous mutants detected among 304 progeny from heterozygous intercrosses (Table 1. Test for one proportion; χ^2^ = 44.74, df = 1, p = 1.124e-11).

**Table 1:** Genotype distribution for *Slc25a21^tm1a(KOMP)Wtsi^* mice.

Allele and Stage	Number of Progeny	Number of Wild-types	Number of Heterozygotes	Number of Homozygotes	Percentage Homozygote Viability
**tm1a E14.5**	35	3	22	8	23%
**tm1a E18.5**	44	10	27	7	16%
**tm1a P14**	304	111	168	25	8%
**tm1b P14**	130	24	68	38	29%
**tm1c P14**	145	26	84	35	24%
**tm1d P14**	193	44	113	36	19%

From heterozygous inter-crossing, homozygous progeny for *Slc25a21^tm1a(KOMP)Wtsi^* were recorded at a sub-Mendelian ratio at post natal day 14 (P14), but a normal ratio at two embryonic time points (E14.5 and E18.5). *Slc25a21^tm1b(KOMP)Wtsi^*, *Slc25a21^tm1c(KOMP)Wtsi^* and *Slc25a21^tm1d(KOMP)Wtsi^* homozygotes were detected at the expected ratio.

**Figure 1:**
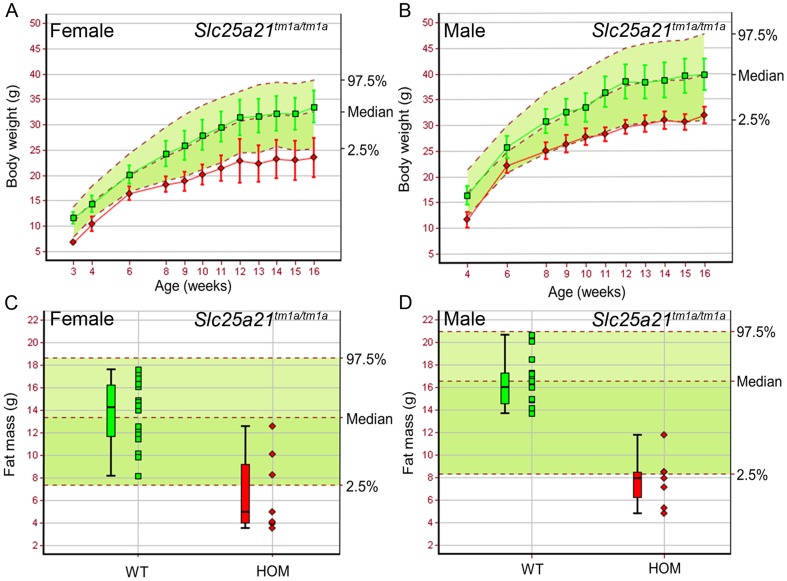
Body weight and fat mass were reduced in homozygous *Slc25a21^tm1a(KOMP)Wtsi^* mice. Homozygous (red symbols) *Slc25a21^tm1a(KOMP)Wtsi^* female (A,C) (n = 7) and male (B,D) (n = 7) mice showed decreased body weight and fat mass compared to local wild-type controls run on the same week (green symbols) (female, n = 22; male, n = 15). Mean (± SD) body weight is plotted against age (A,B). Fat mass is presented as a boxplot, with median, 25^th^ and 75^th^ percentile (box), and the lowest and highest data point within 1.5× inter quartile range (whiskers) shown (C,D). The median and 95% reference range (2.5–97.5^th^ percentiles, dotted lines) for all wild-type mice of the same genetic background and sex (n>750) are displayed on the pale green background.

**Figure 2:**
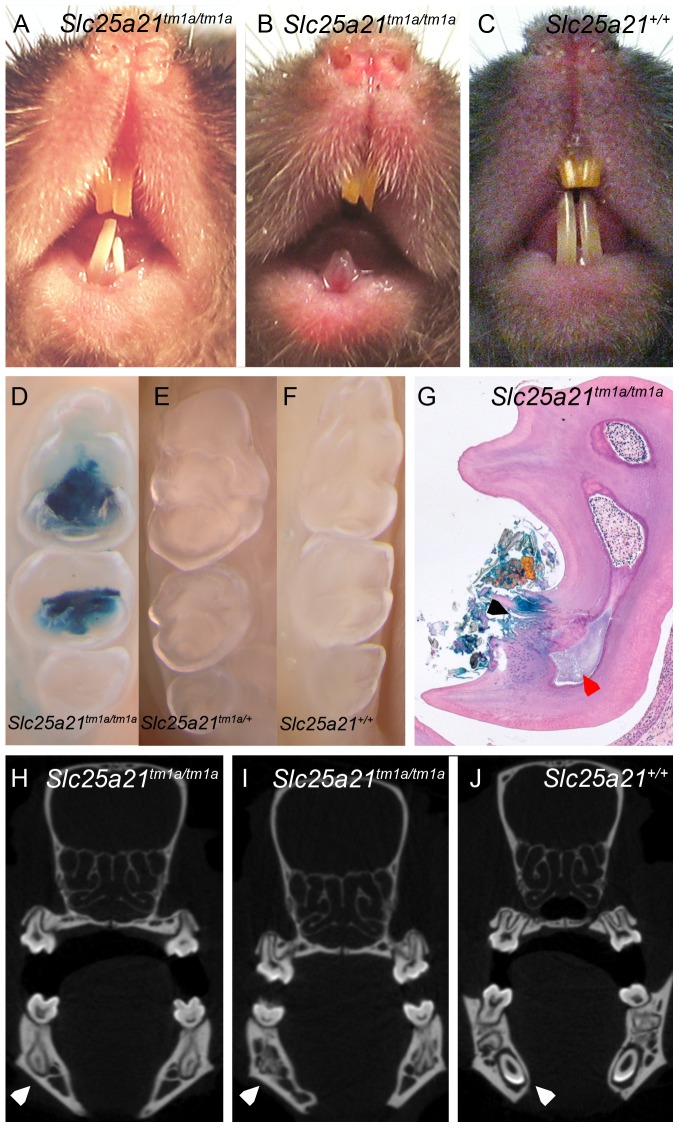
Dental abnormalities observed in homozygous *Slc25a21^tm1a(KOMP)Wtsi^* mice. Dysmorphology analysis revealed underdeveloped, white incisors (A–B) in *Slc25a21^tm1a(KOMP)Wtsi^* homozygous mice as compared to wild-type (C). Wholemount (D–F) and subsequent histological sections (G) demonstrated bacterial *lacZ* activity in cavities within lower jaw molars of *Slc25a21^tm1a(KOMP)Wtsi^* homozygous (D,G) mice compared to heterozygous (E) and wild-type (F) mice. Focal fracturing of the surface dentine is indicated with a black arrow and necrosis of the pulp indicated with a red arrow (G). Micro-CT imaging demonstrated that the root aspect of lower jaw incisors was severely under developed (white arrow) and lacked enamel coating in homozygous *Slc25a21^tm1a(KOMP)Wtsi^* mice (H–I) compared to wild-type (J).

Those homozygous animals surviving to weaning, presented with decreased body weight (Fig. 1A–B), fat mass (Fig. 1C–D) and fat percentage estimate (data not shown), in both sexes, compared to wild-type controls. Furthermore, a range of dental abnormalities were observed in *Slc25a21^tm1a(KOMP)Wtsi^* homozygous mice. At 10 weeks of age 13 out of 14 homozygous *Slc25a21^tm1a(KOMP)Wtsi^* mice were found to have an abnormally shortened snout (data not shown). At 16 weeks of age all fourteen homozygous *Slc25a21^tm1a(KOMP)Wtsi^* mice were found to have macroscopically visible dental abnormalities ranging from white or translucent to severely hypoplastic lower incisors (Fig. 2A–B) compared to wild-type (Fig. 2C). The molars of homozygous *Slc25a21^tm1a(KOMP)Wtsi^* adult mice (Fig. 2D), in particular of the lower jaw, were found to be strongly stained with X-gal (requiring β-galactosidase activity) compared to heterozygous and wild-type (Fig. 2E–F). Microscopic examination of sections from representative animals confirmed that in unaffected areas, the molars were fully developed with normal odontoblast morphology. In affected areas of the molars, there was focal fracturing of the surface dentine and necrosis of the pulp with associated inflammation and bacterial colonies (Fig. 2G). Micro-CT scanning revealed that the lower incisor root area in homozygous *Slc25a21^tm1a(KOMP)Wtsi^* mice was severely under-developed and that the lower incisors appeared to be lacking the enamel coating (Fig. 2H–I) compared to wild-type (Fig. 2J). Furthermore, two dimensional X-ray images revealed several changes of the manidibular morphology including pronounced pogonion, pronounced anterior tuberosity, under-developed incisor process and alveolus (data not shown), potentially indicating a change in the developmental dynamics of the mandible in homozygous *Slc25a21^tm1a(KOMP)Wtsi^* mice. Taken together, the 2D X-ray images, micro-CT and X-gal staining in tooth cavities suggests feeding might be uncomfortable for homozygous *Slc25a21^tm1a(KOMP)Wtsi^* mice which may explain their reduced body weight and fat mass.

**Figure 3:**
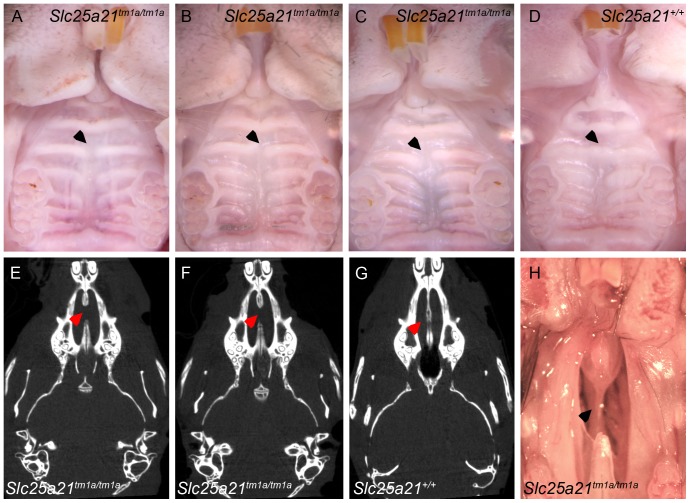
Rugae and vomer abnormalities observed in homozygous *Slc25a21^tm1a(KOMP)Wtsi^* mice. Abnormalities in rugae (in particular number 3) were observed in homozygous *Slc25a21^tm1a(KOMP)Wtsi^* mice (A–C) compared to wild-type (D), indicated with arrows (wt, n = 11; hom, n = 11). Micro-CT analysis revealed that the anterior part of the vomer bone was absent in *Slc25a21^tm1a(KOMP)Wtsi^* homozygous mice (E–F) compared to wild-type (G) (wt, n = 2; hom, n = 12). This was confirmed by dissection of the palate of *Slc25a21^tm1a(KOMP)Wtsi^* mice (H).

Further characterisation uncovered craniofacial abnormalities in adult homozygous *Slc25a21^tm1a(KOMP)Wtsi^* mice. Specifically, they presented with abnormal development of palatal rugae number 3 (Fig. 3A–C) compared to wild-type (Fig. 3D). Micro-CT scanning revealed that the anterior part of the vomer bone was completely absent in homozygous *Slc25a21^tm1a(KOMP)Wtsi^* mice (Fig. 3E–F) compared to wild-type (Fig. 3G), this was confirmed by macroscopic observations (Fig. 3H). The vomeronasal organ (VNO) appeared to be intact. Morphological examination of E18.5 embryo heads from homozygous *Slc25a21^tm1a(KOMP)Wtsi^* mice did not reveal any further craniofacial abnormalities.

**Figure 4:**
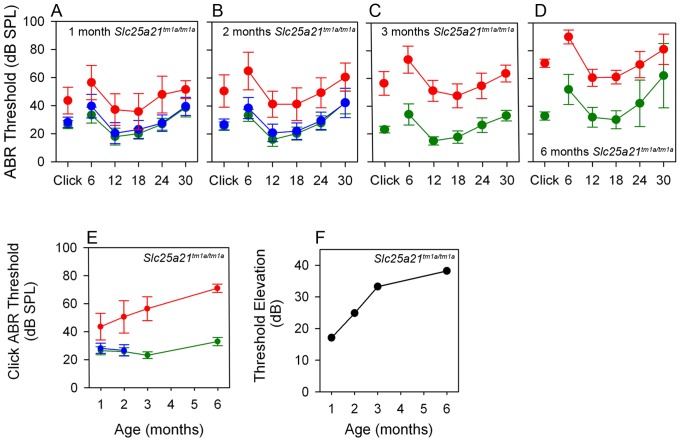
Auditory brainstem response threshold was elevated in *Slc25a21^tm1a(KOMP)Wtsi^* mice. Auditory brainstem response threshold was elevated across all frequencies in homozygous *Slc25a21^tm1a(KOMP)Wtsi^* mice aged 1, 2, 3 and 6 months respectively. Mean ABR thresholds (± SD) are shown for wild-type (green symbols), heterozygote (blue symbols) and homozygote (red symbols) *Slc25a21^tm1a(KOMP)Wtsi^* mice (A–D). The click-evoked ABR thresholds (mean ± SD) plotted as a function of age (months) demonstrated a progressive deterioration in *Slc25a21^tm1a(KOMP)Wtsi^* mice (E); an observation clearly demonstrated by plotting the mean click-evoked ABR threshold elevation of mutants above wild-types as a function of age (months) (F).

Finally, 14 week old *Slc25a21^tm1a(KOMP)Wtsi^* homozygous mice were found to have a hearing impairment as they presented with elevated auditory brainstem response thresholds across all frequencies, with severity of impairment increasing with age (Fig. 4A–D). *Slc25a21^tm1a(KOMP)Wtsi^* homozygous mice displayed an approximately parallel pattern of threshold elevation across the range of frequency stimuli used, a profile which is typical of conductive hearing loss. Fig. 4E shows that click threshold in wild-types was stable with increasing age, at least up to 26 weeks of age, whereas a progressive deterioration was seen in *Slc25a21^tm1a(KOMP)Wtsi^* homozygous mice over the same age range. At 26 weeks of age, some wild-type mice showed age-related high-frequency hearing loss (indicated by larger standard deviations at 24–30 kHz). The thresholds recorded in the *Slc25a21^tm1a(KOMP)Wtsi^* homozygous mice at all ages were significantly elevated above age-matched wild-types (p<0.001) (Fig. 4F).

**Figure 5:**
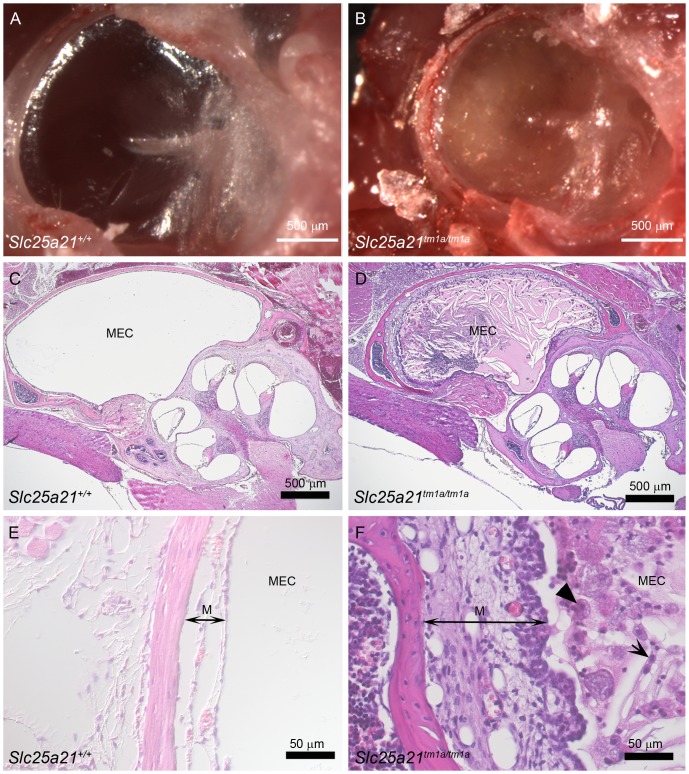
Signs of otitis media in *Slc25a21^tm1a(KOMP)Wtsi^* mice. The tympanic membrane viewed from the external ear canal showed an opaque fluid present in *Slc25a21^tm1a(KOMP)Wtsi^* homozygous mice (B) that was not present in wild-type controls (A). The opaque exudate was clearly visible on haematoxylin and eosin stained histology sections through the middle ear of *Slc25a21^tm1a(KOMP)Wtsi^* homozygous mice (D) compared to wild-types (C). Examination at higher magnification revealed that the exudate containing inflammatory cells including foamy macrophages (solid arrow head), and neutrophils (short arrow) in the middle ear cavity (MEC), and a thickened mucosa (M, double headed arrow) in *Slc25a21^tm1a(KOMP)Wtsi^* homozygous mice due to epithelial hyperplasia, oedema and congestion (F). Wild-type mice appeared normal (E).

To investigate the morphology of the middle ear, the right temporal bone, containing the entire ear, was dissected from *Slc25a21^tm1a(KOMP)Wtsi^* homozygous mice at 9 weeks of age (n = 8) along with wild-type (n = 8) and heterozygous (n = 11) littermates. Compared to wild-types (Fig. 5A), most of the homozygotes (7/8) showed opaque fluid or white debris in the middle ear cavity indicating otitis media (Fig. 5B). Other features of otitis media were more variable, including excessive vascularisation (n = 3) or whitening of the bulla (n = 4) or tympanic membrane (n = 1). The one homozygous *Slc25a21^tm1a(KOMP)Wtsi^* mouse in which exudate was not observed in the middle ear also had ABR thresholds only slightly above those seen in wild-type mice. Heterozygotes had normal middle ears.

The left side temporal bones from the same eight *Slc25a21^tm1a(KOMP)Wtsi^* homozygous mice and wild-type controls were sectioned through the middle ear, stained with haemotoxylin and eosin, and examined microscopically. Inflammatory changes and accumulation of exudate consistent with otitis media were observed in six of the eight homozygotes (Fig. 5D, F).

The middle ear cavity of the six affected *Slc25a21^tm1a(KOMP)Wtsi^* homozygous mice contained variable amounts of an amorphous eosinophilic exudate containing cholesterol crystals, foamy macrophages and neutrophils, indicating an active inflammatory response. In these mice, the lining epithelium of the middle ear cavity was variably hyperplastic and the underlying lamina propria was oedematous with congestion of blood vessels (Fig. 5D, F). None of the littermate controls showed evidence of inflammation (Fig. 5A, C, E).

Of the two *Slc25a21^tm1a(KOMP)Wtsi^* homozygous mice showing little or no effusion one had comparatively normal ABR thresholds, whilst the other had raised ABR thresholds.

The tympanic ring size was normal in *Slc25a21^tm1a(KOMP)Wtsi^* homozygous mice; assessment of micro-CT images revealed no change in adults whilst alizarin red/alcian blue stained embryos revealed no changes at E18.5 (data not shown).

Systematic histopathology assessment of a range of tissues from homozygous *Slc25a21^tm1a(KOMP)Wtsi^* mice did not reveal any further abnormalities, and in particular, no indication of any inflammation other than in the middle ear was found.

### Ablation of *Slc25a21* does not cause signs of 2-oxoadipate acidaemia in mice

It has been proposed that loss of function of ODC, the protein encoded by *SLC25A21*, causes the human disease 2-oxoadipate acidaemia which is clinically characterised by hypotonia, seizures, motor and developmental delay, cerebellar ataxia and varying severities of intellectual disability [13], [14], [15], [16]. Unexpectedly, homozygous *Slc25a21^tm1a(KOMP)Wtsi^* mice did not phenocopy this human disease. In particular, lean mass, grip strength and gait, correlates of the human disease manifestation, were all normal for these mice (data not shown), and no seizures were detected.

**Figure 6:**
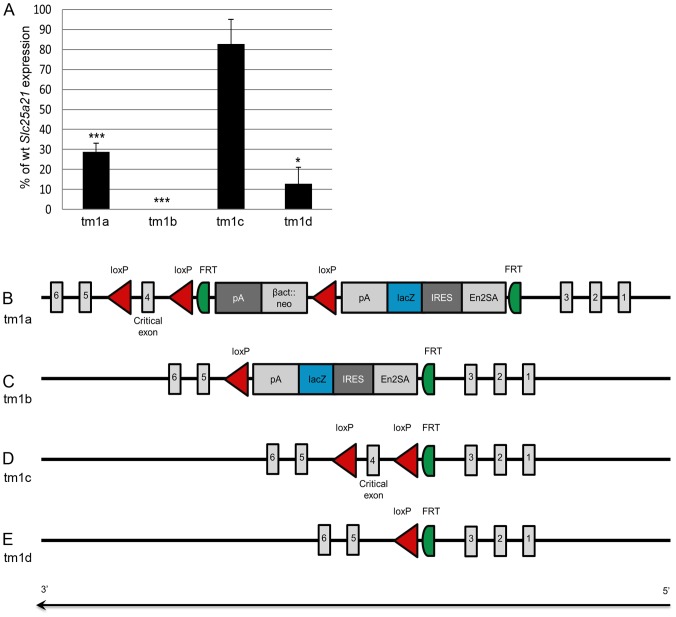
Schematic of the *Slc25a21^tm1(KOMP)Wtsi^* allelic series and the associated RNA expression levels. Quantitative PCR on RNA from E13.5 embryo heads demonstrated that *Slc25a21* RNA expression was significantly reduced in *Slc25a21^tm1a(KOMP)Wtsi^*, *Slc25a21^tm1b(KOMP)Wtsi^* and *Slc25a21^tm1d(KOMP)Wtsi^* homozygotes (* p<0.05; *** p<0.001), but equivalent to wild-type levels in *Slc25a21^tm1c(KOMP)Wtsi^* homozygotes (A). A schematic of the *Slc25a21^tm1(KOMP)Wtsi^* allelic series is presented, consisting of: (B) *Slc25a21^tm1a(KOMP)Wtsi^*, the knockout first reporter (*lacZ*) tagged insertion allele; (C) *Slc25a21^tm1b(KOMP)Wts^* ^i^, the *lacZ* tagged deletion allele generated by breeding with mice ubiquitously expressing Cre recombinase; (D) *Slc25a21^tm1c(KOMP)Wtsi^*, the conditional allele generated by breeding mice carrying the *Slc25a21^tm1a(KOMP)Wtsi^* allele with mice ubiquitously expressing Flp recombinase; and (E) *Slc25a21^tm1d(KOMP)Wtsi^*, the deletion allele arising after breeding mice carrying the *Slc25a21^tm1c(KOMP)Wtsi^* allele with mice ubiquitously expressing Cre recombinase (E).

To ascertain the molecular consequence of the targeting event, expression analysis of *Slc25a21^tm1a(KOMP)Wtsi^* was performed on RNA extracted from embryo heads (E13.5). When using the *Slc25a21* TaqMan assay which spanned exons 8–9, 3′ of the cassette insertion, expression was found to be reduced in homozygous *Slc25a21^tm1a(KOMP)Wtsi^* samples (28.7% of wild-type expression, p = 2.0e-05) but not completely ablated (Fig. 6A). The hypomorphic nature of this allele may have explained the absence of the predicted phenotypes, therefore an allelic series was created (Fig. 6B–E) where tm1b was a *lacZ* reporter tagged deletion with the critical exon (exon 4) removed, tm1c a conditional allele with wild-type function restored, and tm1d the deletion allele where both exon 4 and the *lacZ* reporter were removed. Expression analysis of each of these *Slc25a21* alleles was performed on RNA extracted from embryo heads (E13.5). *Slc25a21* RNA expression was found to be ablated in homozygous *Slc25a21^tm1b(KOMP)Wtsi^* (0% of wild-type expression, expression was below the limit of detection) and greatly reduced in *Slc25a21^tm1d(KOMP)Wtsi^* homozygotes (12.9% of wild-type expression, p = 0.043) (Fig. 6A). As expected, expression levels of *Slc25a21* in embryos homozygous for *Slc25a21^tm1c(KOMP)Wtsi^* were not significantly different from wild-type expression (Fig. 6A).

**Figure 7:**
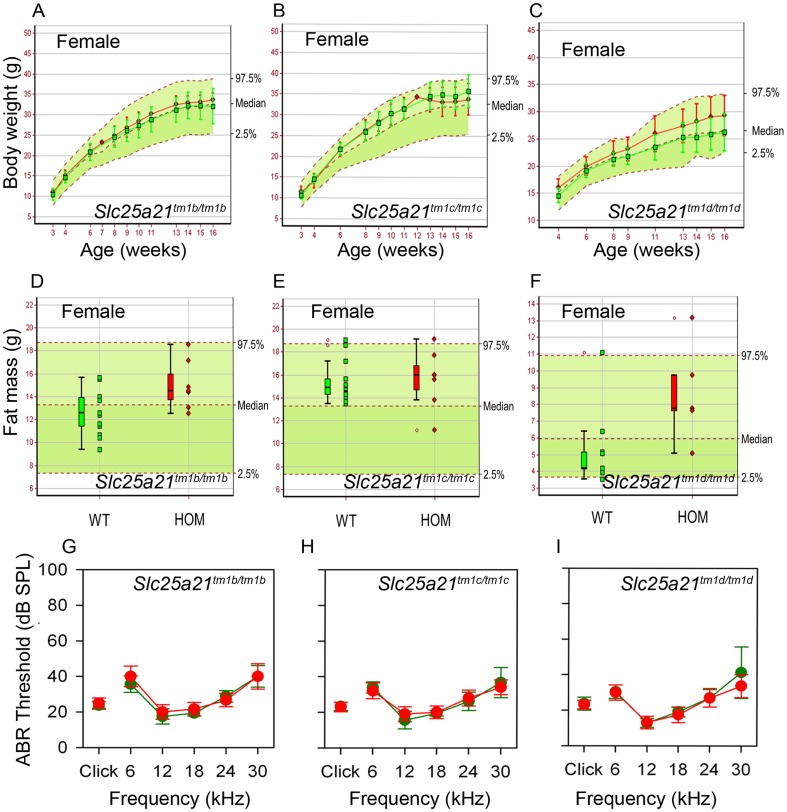
Normal phenotype observed in *Slc25a21^tm1b(KOMP)Wtsi^*, *Slc25a21^tm1c(KOMP)Wtsi^* and *Slc25a21^tm1d(KOMP)Wtsi^* homozygous mice. Body weight and fat mass were normal in females homozygous (red symbols) for *Slc25a21^tm1b(KOMP)Wtsi^* (A,D) (n = 7), *Slc25a21^tm1c(KOMP)Wtsi^* (B,E) (n = 7), and *Slc25a21^tm1d(KOMP)Wtsi^* mice (C,F) (body weight n = 7, fat mass n = 5) compared to local wild-type controls run on the same week (green symbols) (n≥9). Male homozygotes for each of these three alleles and two parameters were also phenotypically normal (data not shown). Mean (± SD) body weight is plotted against age (A–C). Fat mass is presented as a boxplot, with median, 25^th^ and 75^th^ percentile (box), and the lowest and highest data point within 1.5× inter quartile range (whiskers) shown (D–F). The median and 95% reference range (2.5–97.5^th^ percentiles, dotted lines) for all wild-type mice of the same genetic background and sex (n>80) are displayed on the pale green background. ABR thresholds were normal in homozygous *Slc25a21^tm1b(KOMP)Wtsi^* (G), *Slc25a21^tm1c(KOMP)Wtsi^* (H) and *Slc25a21^tm1d(KOMP)Wtsi^* mice (I) (red symbols) compared to wild-type controls (green symbols).

We proceeded by phenotyping animals homozygous for the *Slc25a21^tm1b(KOMP)Wtsi^*, *Slc25a21^tm1c(KOMP)Wtsi^* and *Slc25a21^tm1d(KOMP)Wtsi^* alleles. Following heterozygous intercrossing, homozygous animals for all three alleles were detected at the expected Mendelian ratio. Furthermore, body weight, fat mass and auditory brainstem response thresholds (Mann-Whitney Rank Sum Tests; tm1b, p = 0.12; tm1c, p = 0.48; tm1d, p = 0.59) (Fig. 7A–I) were all found to be normal compared with wild-types, as were the teeth and palatal rugae (data not shown). Full data from the phenotyping pipeline can be viewed online (www.sanger.ac.uk/mouseportal).

These results led us to conclude that the phenotypes observed in mice homozygous for the *Slc25a21^tm1a(KOMP)Wtsi^* targeted allele were not due to ablation of Slc25a21 function, and speculate that off-target effects due to the selection cassette present in the tm1a allele affected expression of neighbouring genes.

### Hypomorphic expression of *Pax9* may account for observed phenotypes

The 1 Mb genomic interval containing *Slc25a21* (500 kb flanking the target gene) is predicted to contain seven known protein coding genes, one non coding RNA, one novel antisense and one putative processed transcript (Fig. S1). Of particular interest is *Pax9*, located at the 3′ end of *Slc25a21* and orientated on the opposite strand. The 3′UTR regions of *Slc25a21* and *Pax9* overlap by 188 bp. Consistent with the phenotypes observed in *Slc25a21^tm1a(KOMP)Wtsi^* homozygous animals, mice carrying a hypomorphic *Pax9* allele had previously been reported as causing reduced viability at weaning [25], and presented with a range of dental and craniofacial abnormalities [25], [26] similar to those described herein.

**Figure 8:**
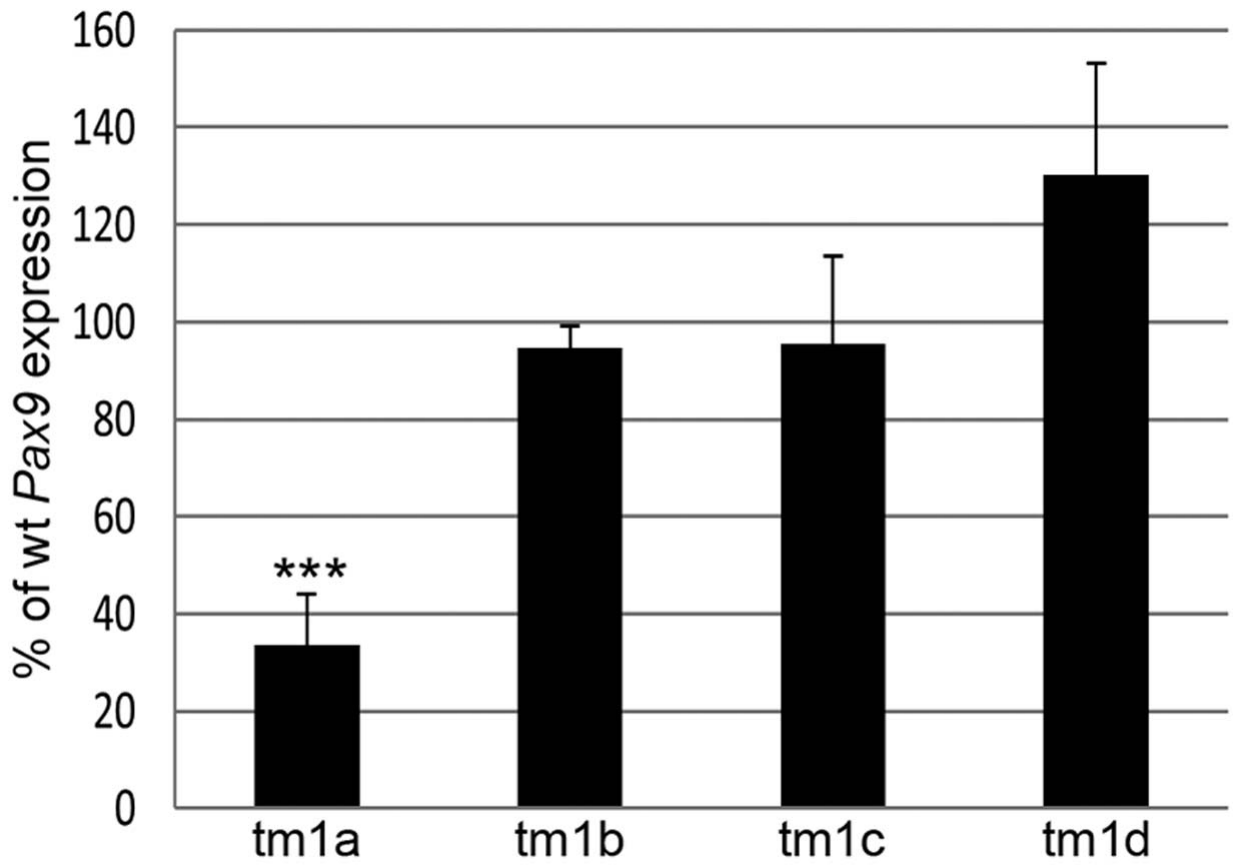
RNA expression of *Pax9* was reduced in *Slc25a21^tm1a(KOMP)Wtsi^* mice. Quantitative PCR on RNA from E13.5 embryo heads demonstrated that *Pax9* RNA expression was significantly reduced in *Slc25a21^tm1a(KOMP)Wtsi^* (*** p = 1.1E-06) homozygotes, but equivalent to wild-type levels in *Slc25a21^tm1b(KOMP)Wtsi^*, *Slc25a21^tm1c(KOMP)Wtsi^* and *Slc25a21^tm1d(KOMP)Wtsi^* homozygotes.

RNA expression analysis of *Pax9* was performed on embryo heads (E13.5) for all four alleles in the *Slc25a21* series. *Pax9* expression was found to be down regulated [approximately 34% of wild-type *Pax9* levels (p = 1.1E-06)] in homozygous *Slc25a21^tm1a(KOMP)Wtsi^* samples (Fig. 8) confirming our hypothesis that we had inadvertently created a *Pax9* hypomorphic allele. *Pax9* levels were found to be unaffected in homozygous *Slc25a21^tm1b(KOMP)Wtsi^*, *Slc25a21^tm1c(KOMP)Wtsi^* and *Slc25a21^tm1d(KOMP)Wtsi^* samples (Fig. 8). A known *Pax9* regulatory element (CNS+6) was located within the targeting construct used to create the *Slc25a21^tm1a(KOMP)Wtsi^* allele [21]. PCR and sequence analysis was used to demonstrate that this sequence was not disrupted during the vector construction or at the homologous recombination stage (Table S1; data not shown). Consequently, we hypothesise that the presence of the promoter driven neomycin cassette in the homozygous *Slc25a21^tm1a(KOMP)Wtsi^* mice caused the reduction of *Pax9* expression.

RNA sequencing was performed to further explore the impact of the targeting event on the genomic environment around *Slc25a21*. Comparing RNA from wild-type and homozygous *Slc25a21^tm1a(KOMP)Wtsi^* E13.5 embryo heads, the only gene in the 1 Mb interval surrounding *Slc25a21* that had significantly altered expression levels was *Pax9* [q (adjusted p value) = 7.10E-06 (data not shown)]. *Pax9* was not found to be differentially expressed in homozygous *Slc25a21^tm1b(KOMP)Wtsi^*, *Slc25a21^tm1c(KOMP)Wtsi^* and *Slc25a21^tm1d(KOMP)Wtsi^* embryo heads. The low endogenous expression level of *Slc25a21* in wild-type mice was below the threshold where significant changes could be detected.

The phenotypes we observed in homozygous *Slc25a21^tm1a(KOMP)Wtsi^* mice were broadly consistent with those reported previously in mice carrying hypomorphic *Pax9* alleles [26] with the exception that the otitis media and hearing impairment reported in this study have not been assessed previously in *Pax9* mutant mice and may represent a novel consequence of *Pax9* suppression.

## Discussion

### Characteristics of 2-oxoadipate acidaemia are absent in mice ablated for *Slc25a21* expression

We report the generation and phenotypic characterisation of the *Slc25a21* allelic series derived from a typical KOMP-CSD allele [3]. Subject to the sensitivity and scope of the phenotyping performed, ablation of *Slc25a21* expression was not found to phenocopy the human disease 2-oxoadipate acidaemia (OMIM 204750) for which it is a candidate gene [11]. There are several possible explanations for this. Whilst no hypotonia, ataxia or seizures were detected in *Slc25a21* null mice [homozygous for the tm1b (KOMP)Wtsi or tm1d (KOMP)Wtsi alleles], a subset of other clinical symptoms associated with 2-oxoadipate acidaemia, including intellectual disability and urine metabolites, were not assessed by the standard Mouse Genetics Project primary phenotyping pipeline with which these animals were screened [2]. Therefore it cannot be ruled out that additional tests, or analysis performed with increased sensitivity, may reveal subtle disease similarities. Alternatively, mitochondrial transport of metabolites is a complex process involving many carrier proteins. Unknown compensatory mechanisms may be at play in *Slc25a21* null mice that prevent the cytoplasmic build-up of oxoadipate and the associated disruption in the catabolism of key amino acids such as lysine [11]. For example, it is known that the substrate specificity of Slc25a21 itself varies between species [11]. Finally, definitive proof of the candidacy of *Slc25a21* for 2-oxoadipate acidaemia is not published and it has been speculated that the disease may be due to defective 2-oxoadipate dehydrogenase, an alternative gene in the same metabolic pathway [11].

### *Slc25a21^tm1a(KOMP)Wtsi^* allele affects expression of a neighbouring gene

The *Slc25a21^tm1a(KOMP)Wtsi^* homozygous mice investigated in this study were found to be sub-viable. Those homozygous animals surviving to weaning presented with growth retardation, orofacial abnormalities, brittle, white incisors and hearing impairment. Given the absence of any detectable phenotype in mice homozygous for the *Slc25a21* tm1b (KOMP)Wtsi, tm1c (KOMP)Wtsi or tm1d (KOMP)Wtsi derived alleles, we conclude that the phenotypes observed in mice homozygous for *Slc25a21^tm1a(KOMP)Wtsi^* were not due to ablation of *Slc25a21* function, and propose that off-target effects of the tm1a allele affected expression of a neighbouring gene. The spatial, temporal and quantitatively correct activity of a gene requires the presence of not only intact coding sequence but also properly functioning regulatory control. Interference of the expression of neighbouring genes by the presence of selectable marker cassettes or short DNA sequences [5], [6], [7], [8], [9] has been reported for genes in close vicinity to the target gene and as far as >100 kb from the targeted gene [6]. Transcriptional silencing of neighbouring genes due to CpG rich sequences within the standard, non-codon optimised, *lacZ* reporter gene used in this KOMP-CSD allele has also been reported [27]. Furthermore, disruption of *cis*-regulatory elements has been linked with abnormal phenotypes in both mice [5] and humans [28], [29]. In addition, a wide variety of mechanisms which cause disruption of gene expression by a change in its chromosomal environment, while leaving the transcription unit intact, have been identified [29].

At E13.5, RNA sequencing revealed that the only gene within the 1 Mb genomic interval surrounding *Slc25a21* that was differentially expressed, compared to wild-type, in homozygous *Slc25a21^tm1a(KOMP)Wtsi^* embryos was *Pax9*, an observation confirmed by quantitative PCR. *Pax9* is located in a 21.06 kb genomic interval directly 3′ of *Slc25a21* and orientated on the opposite strand. Evolutionarily conserved non-coding sequences are present both up-and down-stream of *Pax9*, some of which have been demonstrated to induce *Pax9* expression [21]. One such conserved region, CNS+6, [21] was located within the homology arm of the *Slc25a21^tm1a(KOMP)Wtsi^* targeting construct, and therefore a candidate for causing altered *Pax9* expression. However, sequencing confirmed the integrity of CNS+6 in the *Slc25a21^tm1a(KOMP)Wtsi^* allele. Since homozygous *Slc25a21^tm1b(KOMP)Wtsi^* mice, which still have part of the targeting cassette present (Fig. 6C), were found to be phenotypically normal, interference by the polyA-*β-actin::neo* cassette is the most likely cause of the reduction of *Pax9* expression seen in homozygous *Slc25a21^tm1a(KOMP)Wtsi^* mice. The findings in this study highlight the need to confirm phenotypes found in animals carrying the knockout first conditional ready [tm1a (KOMP)Wtsi or tm1a (EUCOMM)Wtsi] allele by creating and analysing the derived deletion allele. This is a timely reminder, with direct implications on the 3Rs, given the growing resource of knockout first conditional ready targeted ES cells created by the EUCOMM and KOMP initiatives.

### Hypomorphic expression of *Pax9* may account for orofacial abnormalities

The *Pax* family of transcription factors plays a pivotal role in embryonic patterning and disease. The expression of each *Pax* gene is highly regulated in a temporal and spatial manner [30]. During mouse embryogenesis, *Pax9* is expressed mainly in the sclerotome of somites, the pharyngeal pouch endoderm and its derivatives, developing limb buds, and in facial mesenchyme of neural-crest cell origin, including nasal and jaw processes and tooth buds [26], [31]. There is strong expression of *Pax9* in the medial edge epithelium during the critical time points of palate fusion [32]. In adult mice, *Pax9* is expressed in oesophagus and tongue [33]. Looking at published models of *Pax9* deficiency, homozygous *Pax9* knockout mice die shortly after birth and exhibit a wide range of developmental defects [26] including the absence of teeth. Death has been attributed to cleft palate, affecting the maxillary and palatine shelves, resulting in an inability to suckle and respiratory issues [26]. Two hypomorphic alleles of *Pax9* have been published previously [25]. The first, which expressed wild-type *Pax9* mRNA at 7% of control levels, presented with hypoplastic upper incisors lacking enamel, missing lower incisors, unilateral or bilateral absence of the third molar in the upper jaw and the second and third molar in the lower jaw, and the lower molars that were present showed severe attrition. In comparison, the hypomorphic allele with 20% of wild-type *Pax9* mRNA expression relative to controls presented with less severely affected dentition including relatively normal upper incisors, hypoplastic lower incisors lacking enamel and unilateral or bilateral absence of the upper and lower third molar. We now report homozygous *Slc25a21^tm1a(KOMP)Wtsi^* mice which express wild-type *Pax9* mRNA at 34% of control levels and present with an even milder dental phenotype including upper incisors that were commonly maloccluded, which may be due to abnormal snout morphology or the fact that the incisors do not wear against each other. We found fragile, damaged or missing lower incisors which were white or translucent; normally incisors in mice are brownish/yellow due to incorporation of iron-containing pigment in the enamel [34]. The lower incisor root area was severely under-developed. Lower jaw molars showed focal fracturing of the surface dentine and necrosis of the pulp with associated inflammation and bacterial accumulation. The presence of *lacZ* activity within the cavities of the lower molars of homozygous *Slc25a21^tm1a(KOMP)Wtsi^* mice but not heterozygous mice, confirms that bacterial β-galactosidase is the cause of the staining, not expression of the lacZ reporter gene within the targeted allele. Molars in the upper jaw were less affected. The more severe findings in the lower jaw are consistent with findings in the other *Pax9* hypomorphic alleles [25].

Only limited assessment of the palate has been published for the previous *Pax9* hypomorphic lines and no abnormalities were reported [25]. In this study, more extensive analysis was performed and whilst the phenotype was significantly less severe than that reported in *Pax9* null mice [26], abnormalities were identified, characterised largely by discordance of rugae 3 and an incomplete fusion of the vomer bone to the maxilla.

This gradated allelic series of *Pax9* expression levels, extended by the data reported herein, clearly demonstrates that dosage of this gene affects orofacial development and that lower jaw dentition in the mouse is more susceptible to reduced *Pax9* expression [25]. This is consistent with humans where dentition is not uniformly affected by a reduced Pax9 expression [35], [36], [37] and the observation that the minimal *Pax9* gene dosage required for the formation of individual teeth varies, the posterior tooth in each tooth family being the most sensitive to a reduction in *Pax9* levels.

### Otitis media and hearing impairment in mice may be due to disrupted *Pax9* expression

A reduction in the size of the tympanic ring has previously been described in Pax9 mutant mice [26], however the effect on hearing has not previously been assessed. The homozygous *Slc25a21^tm1a(KOMP)Wtsi^* mice have a normal sized tympanic ring but display a clear hearing impairment with increased thresholds at all frequencies tested. The moderate hearing impairment seen in homozygous *Slc25a21^tm1a(KOMP)Wtsi^* mice is robust and reproducible across different cohorts of mice, tested at ages from 4 to 26 weeks of age. The degree of impairment in mutant mice is progressive from 4–14 weeks old, with slower progression to 26 weeks of age. The parallel shifts in audiometric profiles across frequencies at the different ages (Fig. 4) are consistent with an underlying conductive hearing loss. Middle ears of the majority of homozygous *Slc25a21^tm1a(KOMP)Wtsi^* mice were filled with fluid of varying viscosity at all ages examined and sections revealed inflammation of the lining of the middle ear. The presence of eosinophilic exudate and the thickening of the mucosa are evident in the homozygous *Slc25a21^tm1a(KOMP)Wtsi^* mice, indicating an on-going inflammatory process. Whilst the aetiology of otitis media is complex [38] and was beyond the scope of this study, the correlation between signs of otitis media and elevated ABR thresholds supports our suggestion that the otitis media is a major contributor to the hearing impairment observed in the young adult homozygous *Slc25a21^tm1a(KOMP)Wtsi^* mice. However, cochlear dysfunction cannot be ruled out especially in the 26 week-old group of both mutants and controls. ABR data from *Slc25a21^tm1b(KOMP)Wtsi^*, *Slc25a21^tm1c(KOMP)Wtsi^* and *Slc25a21^tm1d(KOMP)Wtsi^* homozygotes indicate that hearing thresholds are normal.

## Conclusion

In conclusion, ablation of *Slc25a21* expression was not found to phenocopy the human disease 2-oxoadipate acidaemia (OMIM 204750) for which it is a candidate gene. The phenotypes observed in mice homozygous for *Slc25a21^tm1a(KOMP)Wtsi^* were not due to ablation of *Slc25a21* function, but instead the presence of the selection cassette affected expression of the neighbouring gene, *Pax9*. The resulting mutant line confirmed and extended the existing knowledge of *Pax9* gene dosage effect on orofacial development and represents a novel model of otitis media that may be due to reduced *Pax9* expression.

## Supporting Information

Figure S1 **Ensembl view of 1 Mb genomic interval encompassing** ***Pax9*** **and** ***Slc25a21*** **.** Ensembl view of the 500 kb of genomic DNA flanking the 5′ and 3′ end of *Slc25a21*. The *Slc25a21^tm1a(KOMP)Wtsi^* targeting construct (shown in blue) designates exon 4 as the critical exon.
(TIF)

File S1 **Supplementary materials.**
(DOC)

Table S1 **The sequence of primers used for molecular characterisation.** Molecular characterisation of the targeting event was performed using a combination of PCR assays. The 5′ to 3′ primer sequence along with the expected product size (bp) is presented.
(DOC)

Table S2 **Comparison of the outcomes from automatic data evaluation and manual assessment.** Comparison of the outcomes from automatic data evaluation and manual assessment for each parameter included in the dataset is presented. Discrepancies between these two methods of assessment are highlighted, and the rationale behind the manual assessment is provided in each instance there was a discrepancy.
(DOC)
