# Supplementary figures and images for: Targeting of Slc25a21 Is Associated with Orofacial Defects and Otitis Media Due to Disrupted Expression of a Neighbouring Gene
Source: PLoS One. 2014 Mar 18;9(3):e91807. doi: 10.1371/journal.pone.0091807 (PMC3958370; doi:10.1371/journal.pone.0091807)

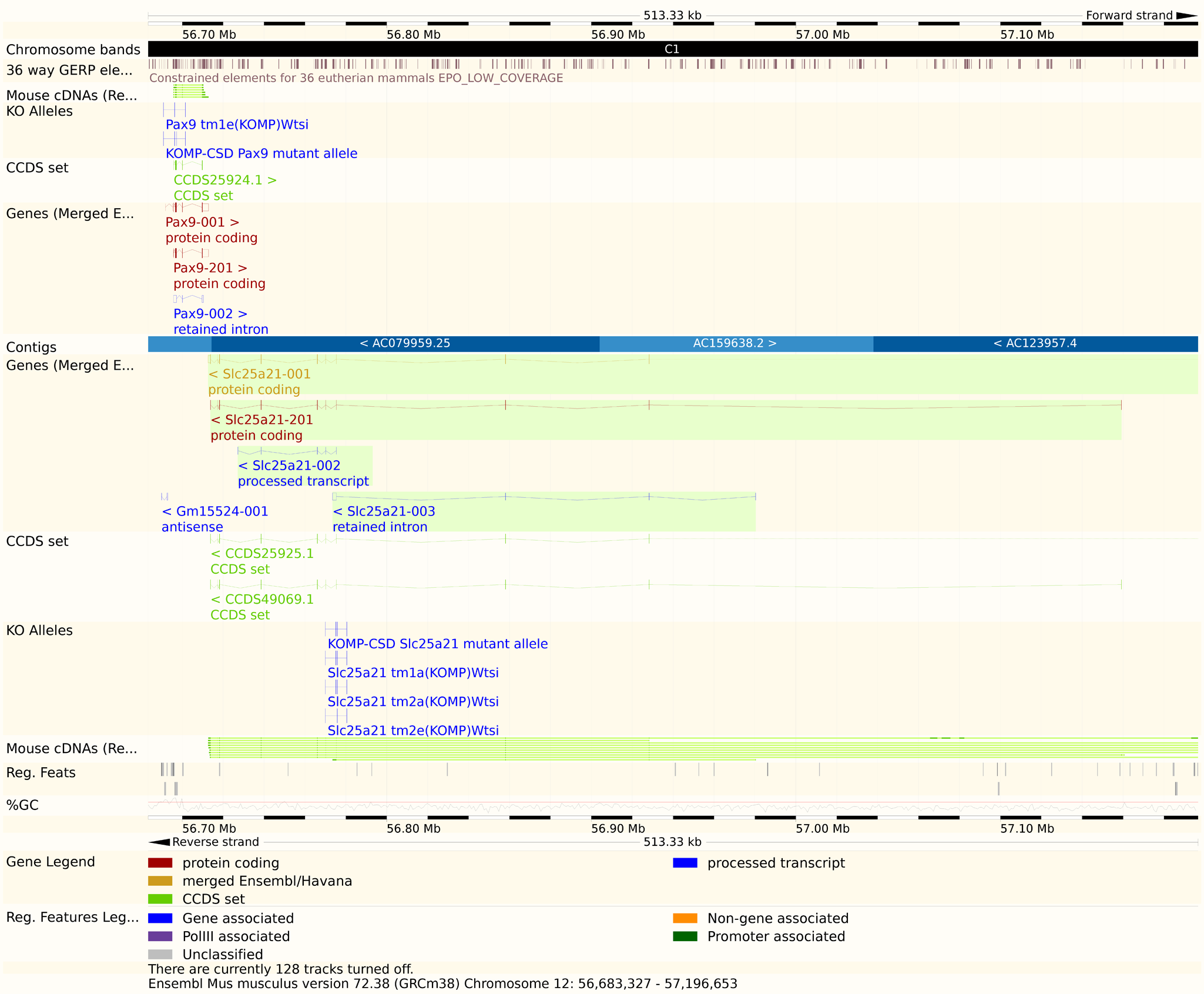

Supplement: Figure S1 — Ensembl view of 1 Mb genomic interval encompassing Pax9 and Slc25a21 . Ensembl view of the 500 kb of genomic DNA flanking the 5′ and 3′ end of Slc25a21. The Slc25a21tm1a(KOMP)Wtsi targeting construct (shown in blue) designates exon 4 as the critical exon. (TIF) [file pone.0091807.s001.tif]
