# Supplementary material for: Targeting of Slc25a21 Is Associated with Orofacial Defects and Otitis Media Due to Disrupted Expression of a Neighbouring Gene
Source: PLoS One. 2014 Mar 18;9(3):e91807. doi: 10.1371/journal.pone.0091807 (PMC3958370; doi:10.1371/journal.pone.0091807)
Supplement: File S1 — Supplementary materials. (DOC) [file pone.0091807.s002.doc]

**Supplementary Material**

**Validation of targeting event**

To confirm the integrity and validity of each allele studied, detailed molecular characterisation was performed using; a) 5’ and 3’ long range PCR (LRPCR) (Supplementary Table 1) with sequencing of products to verify they amplified a region outside the homology arms; b) loss of an amplified wild-type (wt) specific band in homozygotes (hom) compared to controls; and c) failure to amplify a product from an assay designed to detect integration of the vector backbone. The integrity of the targeted allele was verified by a number of short range PCR assays to detect the 5’ *FRT* site, the *lacZ* cassette, and the presence of the *loxP* site downstream of the critical exon. A quantitative PCR (qPCR) assay to detect the copy number of the neomycin selection marker (*neo*) was also used to check for multiple integrations. For more details see Ryder et al., 2013 .

**Expression analysis by quantitative PCR**

E13.5 embryo heads [*Slc25a21^tm1a(KOMP)Wtsi^* (wt, n=4; hom, n=4), *Slc25a21^tm1b(KOMP)Wtsi^* (wt, n=4; hom, n=3), *Slc25a21^tm1c(KOMP)Wtsi^* (wt, n=4; hom, n=3), and *Slc25a21^tm1d(KOMP)Wtsi^* (wt, n=4; hom, n=3)] were processed for expression analysis by qPCR. Samples were collected and stored at -20°C in RNAlater (Ambion, Paisley, UK) until required. Frozen tissue was homogenized using a Qiagen Tissuelyser (Qiagen, Crawley, UK) and RNA extracted using a Qiagen fibrous tissue extraction kit (Qiagen). 1ul (~300ng) of RNA was used in a 10ul reaction using an RNA-to-CT One Step kit (Applied Biosystems, Warrington, UK). A custom FAM-labelled TaqMan assay (Applied Biosystems) spanning the junction of exons 8-9, 3’ to the floxed exon (Slc25a21_E8-9_F: CTGCTTCAAAACAATGGAGATGAT, Slc25a21_E8-9_R: GGGACCAGGCCTTTGTATAAGG, Slc25a21_E8-9_M: CGGGAAGAAGGGATTT) was used in a multiplex reaction with a *Gapdh* endogenous control (4352339E, Applied Biosystems) or with *B2m* endogenous control (Mm00437762_m1, Applied Biosystems). For *Pax9* expression analysis, a pre-designed TaqMan assay was used (Mm00440629_m1, Applied Biosystems). Reactions were performed in triplicate and amplified on an ABI Viia7 qPCR machine. Analysis was performed on Viia7 1.1 software using the relative quantification module. RQ values were calculated using the 2^-ddCt^ method ; the average dCt of the wild-type animals for each allele was used as the calibration value to calculate the ddCt values for the mutants .

**RNA sequencing**

cDNA libraries were created using 5μg of total RNA from a subset of the above E13.5 embryo head samples [3 homozygotes and 2 wild-types for each allele (12 mutant and 8 control samples in total)] as follows. Using a TruSeq RNA Sample Prep v2 kit (Illumina), poly-A tailed RNA (mRNA) was purified from total RNA using an oligo dT magnetic bead pull-down. The resulting mRNA was fragmented using metal ion-catalyzed hydrolysis and random priming used to synthesis double-stranded cDNA.  End repair was performed with a combination of fill-in reactions and exonuclease activity to produce blunt ends. A single “A” base was added to blunt ends followed by ligation to Illumina Paired-End Sequencing adapters containing unique index sequences, allowing samples to be pooled. The resulting libraries were amplified through 10 cycles of PCR using KAPA Hifi Polymerase, products were pooled based on a post-PCR Agilent Bioanalyzer, then the pool was size-selected using the LabChip XT Caliper (200-300bp range). The multiplexed library was sequenced on the Illumina HiSeq 2000 (75bp paired-end read length) aiming for >3Gigabases of data per sample. RNA-seq analysis was conducted using the STAR read aligner , with quantification of RNA abundance and analysis of differential expression using cuffdiff2 . Cuffdiff2 was run with a mask file to exclude tRNA and rRNA regions known to the UCSC genome browser rmsk table. The gene annotation file used was gained from the mm10 UCSC build of the illumina iGenomes distribution .

**Physical assessment, body composition, radiography and clinical chemistry**

Assays of particular relevance from the standard Sanger Institute Mouse Genetics Project phenotyping screen are described below.

Body weight was collected at regular intervals. Gross morphological observations were collected at 10 weeks of age using a standardised list of parameters, including snout and incisor morphology. High resolution radiographs were collected at 14 weeks of age under anaesthesia with Ketamine hydrochloride (100mg/kg, Ketaset, Fort Dodge Animal Health, Southampton, UK) and Xylazine hydrochloride (10mg/kg, Rompun, Bayer Animal Health, Newbury, UK) using a Faxitron X-ray cabinet (MX-20, Faxitron X-ray Corp., Wheeling, IL, USA) and assessed using a standard set of parameters, including skull shape, mandible and teeth. Whilst anaesthetised, body composition [fat mass (g), fat percentage estimate (%), lean mass (g), bone mineral density (g/cm^2^) and bone mineral content (g)] was assessed by densitometry using a PIXImus II Imager (GE Lunar, Madison, WI, USA). Reversal agent was administered following completion of recording (1mg/kg, Antisedan, Pfizer, Tadworth, UK).

At 16 weeks of age, non-fasted mice were anaesthetised with Ketamine/Xylazine as above and blood collected into heparinised paediatric tubes (Kabe Labortechnik GmbH, Numbrecht, Germany) via the retro-orbital sinus. Plasma was analysed for 27 parameters, including metabolic, liver, muscle and kidney panels, electrolytes, minerals and iron, pancreatic enzyme and protein parameters (Olympus AU400, Beckman Coulter Ltd, High Wycombe, UK).

**Reporter gene analysis**

*LacZ* reporter gene wholemount expression analysis was performed on *Slc25a21^tm1a(KOMP)Wtsi^* mice aged 6-12 weeks (wt, n=2; het, n=3; hom, n=9) as described previously . In brief, mice were fixed by cardiac perfusion using 4% paraformaldehyde (PFA; Electron Microscopy Sciences, Hatfield, UK). Following dissection, the tissues to be stained were fixed an additional 30 min in 4% PFA. The tissues were rinsed in PBS and transferred to X-gal staining solution (0.1% 5-bromo-4-chloro-indolyl-β-D-galactopyranoside, Invitrogen, Paisley, UK) for up to 48 hours at 4^o^C. All solutions used prior to and including the staining step were at pH 8. After an additional overnight post-staining fixation in 4% PFA, tissues were cleared with 50% glycerol (VWR, Radnor, USA) then transferred to 70% glycerol with sodium azide (Sigma, St Louis, USA) for long-term storage. Tissues were reviewed and imaged (MZ16A dissecting microscope, Leica, Wetzlar, Germany; DFC490 digital camera, Canon Powershot G5, Japan). Some tissues were decalcified, embedded in paraffin wax, sectioned at 8μm and stained with haematoxylin and eosin.

**Bone and cartilage staining**

Bone and cartilage staining of *Slc25a21^tm1a(KOMP)Wtsi^* E18.5 embryos (wt, n=10; het, n=27; hom, n=12) was performed using a protocol based on the Cold Spring Harbour method for Alcian blue/Alizarin Red staining . Briefly, after fixation in 95% ethanol for 48 hours, ethanol was replaced with ≥99.5% acetone for a further 48 hours (Sigma, St Louis, USA) to remove fat from the embryo. Embryos were transferred to 0.015% Alcian Blue (Sigma Aldrich, Gillingham UK) for 24 hours, washed several times with 95% ethanol, then cleared overnight with 1% KOH (Sigma, St Louis, USA) after which they were stained with 0.005% Alizarin Red (Sigma Aldrich, Gillingham UK) for 2 hours. After further clearing in 1% KOH, embryos were dehydrated with increasing concentrations of glycerol before being stored in the dark in 70% glycerol. Embryos were reviewed and imaged as described above.

**Data analysis and statistics**

The Sanger Institute Mouse Genetics Project primary phenotyping screen incorporates control animals run on the same week, but not necessarily the same day, as mutants. For this reason, use of inferential statistics is not advisable. A reference range is intended to encompass all sources of variation seen in our pipeline, including operator and day-to-day variation. The reference range is a conservative method that does not yield a p-value. It relies on establishing natural variation in the parameters analysed, which is possible for a high throughput project where large wild-type data sets are generated and available to build a robust reference range. For each mouse line, typically, the Sanger Institute Mouse Genetics Project analyses 7 mutant mice per sex, and >60% (5/7) of them must lie either above or below the reference range for a significant call to be automatically assigned. In this scenario, the probability of false positives is ~ 6×10^−6^ per sex per variable tested . Time course data had an additional layer to the analysis; each time point was assessed for significance using the same rules as for continuous data, then if either three or more sequential time points were significant or more than 40% of all time points were significant, the mutant line was said to have a significant phenotype. For categorical variables, a Fisher’s exact test was used to identify a statistically significant difference between the ratio of possible outcomes observed in mutant mice compared with that observed in a cumulative baseline built from the data arising from controls from the same genetic background, age and sex. To assess biological significance a second filter was then applied that looked for a ≥70% change in the phenotype assignment for that variable within each genotype/sex group. The automatic identification of significant calls using the above rules was complemented by a manual assessment made by a biological expert who used knowledge of events on the day, or across sexes, or related variables, to highlight additional potentially abnormal phenotypes (Supplementary Table 2). It may be the case that the appearance of a phenotype within the mutant population is seen so rarely within the baseline wild-type population that significance of that phenotype, although not fully penetrant within the mutant population, is warranted.

**Supplementary Material References**
