# Supplementary material for: Targeting of Slc25a21 Is Associated with Orofacial Defects and Otitis Media Due to Disrupted Expression of a Neighbouring Gene
Source: PLoS One. 2014 Mar 18;9(3):e91807. doi: 10.1371/journal.pone.0091807 (PMC3958370; doi:10.1371/journal.pone.0091807)
Supplement: Table S1 — The sequence of primers used for molecular characterisation. Molecular characterisation of the targeting event was performed using a combination of PCR assays. The 5′ to 3′ primer sequence along with the expected product size (bp) is presented. (DOC) [file pone.0091807.s003.doc]

**Supplementary Table 1. The sequence of primers used for molecular characterisation.**

| **Assay** | **Forward Primer** | **Reverse Primer** | **Product size (bp)** |
| --- | --- | --- | --- |
| **5' LRPCR** | GTGTGTGAGCTCACATGTGTGTTCCAGTTC | CACAACGGGTTCTTCTGTTAGTCC | 5076 |
| **3' LRPCR** | CACACCTCCCCCTGAACCTGAAAC | CTACATGCAATCTTCTAAGCAATTCAC | 8014 |
| **tm1a WT** | CAATGCTGACATCGAGGGAC | GGTGGCTAAGTGATCCTGGG | 416 |
| **tm1a MUT** | CAATGCTGACATCGAGGGAC | TCGTGGTATCGTTATGCGCC | 148 |
| **tm1a *lacZ*** | ATCACGACGCGCTGTATC | ACATCGGGCAAATAATATCG | 108 |
| **tm1b conversion** | CGGTCGCTACCATTACCAGT | ACTGATGGCGAGCTCAGACC | 380 |
| **tm1c conversion** | AAGGCGCATAACGATACCAC | CCGCCTACTGCGACTATAGAGA | 218 |
| **tm1d conversion** | AAGGCGCATAACGATACCAC | ACTGATGGCGAGCTCAGACC | 174 |
| **CNS+6** | GGGAACAAGAAGCCTTCACA | TCCTTTCCAACTTTATGCCATT | 1458 |

Molecular characterisation of the targeting event was performed using a combination of PCR assays. The 5’ to 3’ primer sequence along with the expected product size (bp) is presented.
