# Supplementary material for: Targeting of Slc25a21 Is Associated with Orofacial Defects and Otitis Media Due to Disrupted Expression of a Neighbouring Gene
Source: PLoS One. 2014 Mar 18;9(3):e91807. doi: 10.1371/journal.pone.0091807 (PMC3958370; doi:10.1371/journal.pone.0091807)
Supplement: Table S2 — Comparison of the outcomes from automatic data evaluation and manual assessment. Comparison of the outcomes from automatic data evaluation and manual assessment for each parameter included in the dataset is presented. Discrepancies between these two methods of assessment are highlighted, and the rationale behind the manual assessment is provided in each instance there was a discrepancy. (DOC) [file pone.0091807.s004.doc]

**Supplementary Table 2. Comparison of the outcomes from automatic data evaluation and manual assessment**

| **Test** | **Parameter** | **Auto call** | **Manual call** | **Rationale of manual assessment** |
| --- | --- | --- | --- | --- |
| Weight curve 4-16wks (Male) | Body weight | Not significant | Significant | Manual call is supported by trend observed in male data that is consistent with the decreased body weight observed in females. |
| Weight curve 4-16wks (Female) | Body weight | Significant | Significant | Manual and Auto-call consistent. |
| X-ray imaging (Male) | Mandible | Not significant | Significant | Manual call is based on the appearance of a phenotype that, although observed at a low frequency in the mutant, is rarely observed in the baseline wild-type population. This observation is also supported by micro CT analysis and the nature of the orofacial phenotype. |
| X-ray imaging (Female) | Mandible | Not significant | Not significant | Manual and Auto-call consistent. |
| Body composition DEXA (Male) | Fat mass | Not significant | Significant | Manual call is based on mutant data being clustered across the periphery and outside the boundaries of the reference range. The Auto-call rule is considered too stringent on this occasion. Manual call is also supported by decreased fat mass in the females. |
| Body composition DEXA (Female) | Fat mass | Not significant | Significant | Manual call is based on mutant data being clustered across the periphery and outside the boundaries of the reference range. The Auto-call rule is considered too stringent on this occasion. Manual call is also supported by decreased fat mass in the males. |
| Body composition DEXA (Male) | Fat percentage estimate | Not significant | Significant | Manual call is based on mutant data being clustered across the periphery and outside the boundaries of the reference range. The Auto-call rule is considered too stringent on this occasion. Manual call is also supported by decreased fat percentage estimate in females. |
| Body composition DEXA (Female) | Fat percentage estimate | Not significant | Significant | Manual call is based on mutant data being clustered across the periphery and outside the boundaries of the reference range. The Auto-call rule is considered too stringent on this occasion. Manual call is also supported by decreased fat percentage estimate in males. |
| Gross morphological assessment (Male) | Snout morphology | Significant | Significant | Manual and Auto-call consistent. |
| Gross morphological assessment (Female) | Snout morphology | Significant | Significant | Manual and Auto-call consistent. |
| Gross morphological assessment (Male) | Incisors | Not significant | Significant | Manual call is based on the appearance of a phenotype that, although observed at a low frequency in the mutant, is rarely observed in the baseline wild-type population. Manual call is also supported by observations of the teeth in females and macroscopic analysis at 16 weeks of age. |
| Gross morphological assessment (Female) | Incisors | Not significant | Significant | Manual call is based on the appearance of a phenotype that, although observed at a low frequency in the mutant, is rarely observed in the baseline wild-type population. Manual call is also supported by observations of the teeth in males and macroscopic analysis at 16 weeks of age. |
